# Naturally occurring antisense RNA of histone H2a in mouse cultured cell lines

**DOI:** 10.1186/1471-2156-6-23

**Published:** 2005-05-14

**Authors:** Hiromi Nishida, Yasuhiro Tomaru, Yuko Oho, Yoshihide Hayashizaki

**Affiliations:** 1 Laboratory for Genome Exploration Research Group, RIKEN Genomic Sciences Center (GSC), RIKEN Yokohama Institute, 1-7-22 Suehiro-cho, Tsurumi-ku, Yokohama, Kanagawa 230-0045, Japan

## Abstract

**Background:**

An antisense transcript of histone H2a that has no significant protein-coding region has been cloned from a mouse full-length cDNA library. In the present study, we evaluated this transcript by using RT-PCR and compared the expression patterns of the sense and antisense transcripts by using quantitative RT-PCR (qRT-PCR).

**Results:**

This antisense RNA was expressed in three mouse cell lines. We call it ASH2a. ASH2a includes not only the complementary sequence of the transcript of *Hist2h2aa2 *(a replication-dependent histone H2a gene), but also that of the promoter of *Hist2h2aa2*. The upstream genomic sequence of the transcription start site of the ASH2a-coding gene (*ASH2a*) lacks both CCAAT and TATA boxes. This absence suggests that the regulation of *ASH2a *is different from that of the replication-dependent histone H2a genes. Findings from qRT-PCR indicated that the expression pattern of *ASH2a *was different from that of *Hist2h2aa2*. Expression of *Hist2h2aa2 *peaked at 2 to 4 h during S-phase, but that of *ASH2a *peaked at 1 h.

**Conclusion:**

We showed the existence of ASH2a, a histone H2a antisense RNA, in mouse cultured cells. The expression pattern of ASH2a is different from that of the sense RNA.

## Background

A comprehensive search of the Functional Annotation of Mouse (FANTOM) database revealed about 30000 full-length cDNA clones without a significant protein-coding region [1]. Indeed, antisense transcripts seem to be present in 10% to 20% of genes in human and mouse genomes [2-6]. These findings suggest that many biological reactions related to antisense transcripts and/or protein-noncoding transcripts are still unrevealed [7]. On the other hand, the cDNA database sometimes includes reverse complements of real transcripts. These artifacts are excluded from the database on the basis of the sequences of intron-splicing sites. Therefore, transcripts without any introns need more experimental evaluation than computational annotation to ascertain their validity.

Histone mRNAs regulated by the cell cycle increase at the beginning of S-phase and decrease at the end of S-phase [8]. Among 20 histone H2a-coding genes, 18 are replication-dependent; the other 2 are replication-independent [9]. The replication-dependent genes lack introns and a poly (A) signal and contain a highly conserved stem-loop structure at the 3' end of the mRNA. This stem-loop structure plays an important role in mRNA processing and stability [10-12]. The promoters of the replication-dependent histone genes contain CCAAT and TATA boxes [13].

As far as we know, *Drosophila *histone H3 antisense [14] and *Leishmania *histone H1 antisense [15] transcripts have been reported, but no histone H2a antisense RNA or mammalian histone antisense RNA has been reported. FANTOM 2 [1] contains an antisense transcript (FANTOM clone ID 2210403F13; accession number AK028129) of histone H2a. We call this antisense transcript ASH2a. Comparison of the nucleotide sequence of ASH2a and the mouse genome sequence showed that the ASH2a-coding gene (*ASH2a*) lies on chromosome 6 without introns. The sequence of ASH2a is exactly complementary to that of the coding region of *Hist2h2aa2 *(a replication-dependent histone H2a gene) and that of the promoter. In the present study, we evaluated *ASH2a *transcript by using RT-PCR and compared the expression patterns of *Hist2h2aa2 *and *ASH2a *by using qRT-PCR.

## Results

### Detection of ASH2a

**Figure 1:**
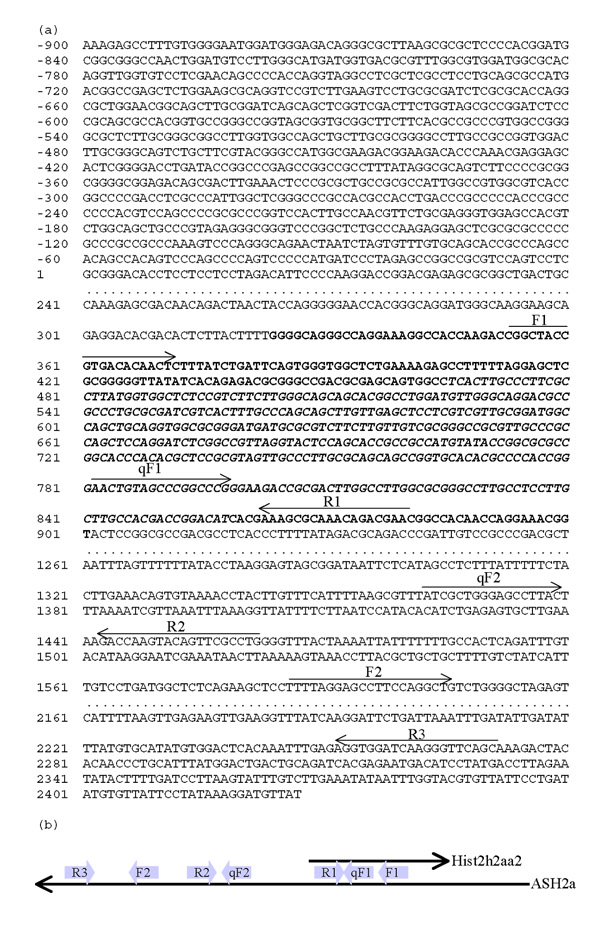
(a) Nucleotide sequence of *ASH2a*. *ASH2a *is encoded from positions 1 to 2427. Bold characters indicate overlap with the *Hist2h2aa2 *transcript (italic = protein-coding region). Arrows indicate primers used in this study. (b) Relationship between *Hist2h2aa2 *and *ASH2a *RNAs. Arrows indicate the locations of the primers.

**Figure 2:**
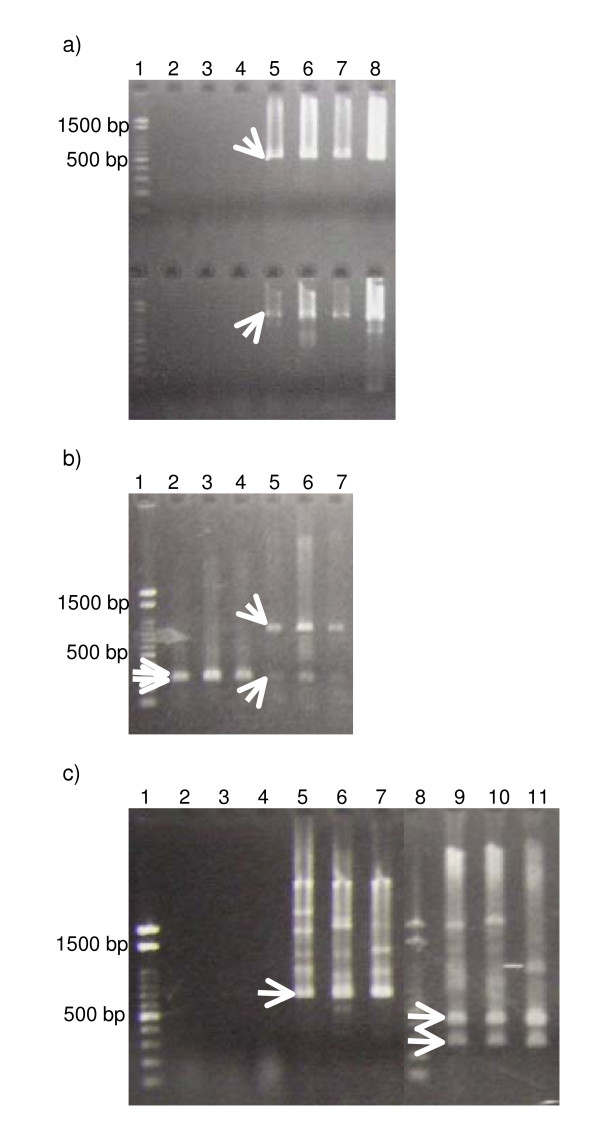
(a) RT-PCR products from cDNAs obtained by priming total RNA with random hexamers. Lanes: 1, DNA ladder (100-bp ladder, TOYOBO); 2–7, RT-PCR products amplified with primers F1 and R1 (upper) and those amplified with primers F1 and R2 (lower). RNA was extracted from Hepa 1–6 (lanes 2 and 5), 3T3 (lanes 3 and 6), and LLC (lanes 4 and 7) cells. Superscript III was not added in the reaction of lanes 2–4. Lane 8, PCR product of genomic DNA amplified with primers F1 and R1 (upper) and that amplified with primers F1 and R2 (lower). Arrows indicate the expected products. (b) Patterns of digestion of PCR products by *Pst*I. Lanes: 1, DNA ladder; 2–4, digests of PCR products amplified with primers F1 and R1. PCR product was produced from Hepa 1–6 (lane 2), 3T3 (lane 3), and LLC (lane 4). Lanes 5–7, digests of PCR products amplified with primers F1 and R2. PCR product was produced from Hepa 1–6 (lane 5), 3T3 (lane 6), and LLC (lane 7). Arrows indicate the expected products. (c) RT-PCR products and the *Eco*RI-digest patterns of cDNAs obtained by priming total RNA with the specific primer R3. Lanes: 1 and 8, DNA ladder; 2–7, RT-PCR products amplified with primers F2 and R3. RNA was extracted from Hepa 1–6 (lanes 2 and 5), 3T3 (lanes 3 and 6), and LLC (lanes 4 and 7). Superscript III was not added in the reaction of lanes 2–4. Lanes 9–11, *Eco*RI-digests of PCR products amplified with primers F2 and R3. PCR product was produced from Hepa 1–6 (lane 9), 3T3 (lane 10), and LLC (lane 11). Arrows indicate the expected products.

First, cDNAs were synthesized by using the random hexamer oligonucleotide for RNAs from the Hepa 1–6, 3T3, and LLC cell lines. RT-PCR was performed for two targets, the sense-antisense overlap region (between F1 and R1 in Fig. 1) and the overlap region plus the antisense-unique region (between F1 and R2 in Fig. 1). All PCR products for both targets had expected sizes (Fig. 2a). To check the PCR products, we digested them with *Pst*I (Fig. 2b). All digests of the PCR products for the region had the expected sizes (251 and 267 bp for the sense-antisense overlap regions; 251 and 858 bp for the overlap region plus the antisense-unique regions).

Second, to elucidate the expression of *ASH2a*, we transcribed the first-strand cDNA with primer R3, which hybridizes specifically to ASH2a. A product of the expected size (685 bp) was obtained (Fig. 2c). In addition, *Eco*RI digested the PCR product to 269- and 416-bp fragments (Fig. 2c). These results are consistent with the nucleotide sequence of *ASH2a*.

### Quantitative RT-PCR

**Figure 3:**
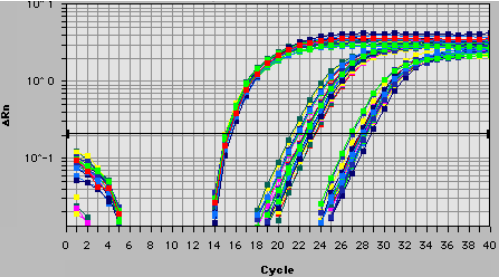
Representative amplification plot. Curves indicate amplification from transcripts of *GAPDH *(first group of rising curves), *Hist2h2aa2 *(second), and *ASH2a *(third). Different colors indicate that each result from 0 h to 12 h (13 points). X-axis, cycle numbers; Y-axis, ΔRn.

**Figure 4:**
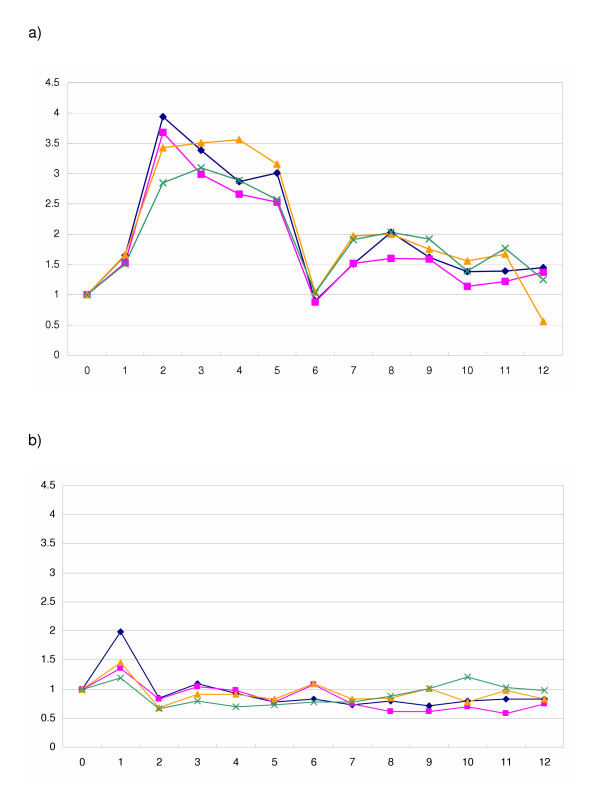
Transcript expression patterns. (a) Transcripts of *Hist2h2aa2*. (b) Transcripts of *ASH2a*. X-axis, time (hours); Y-axis, relative expression level, adjusted to 1.0 at 0 h. The qRT-PCR analyses were performed 4 times (indicated by different colors).

Because the sequence of the *Hist2h2aa2 *transcript is not unique, being completely overlapped by the ASH2a sequence, the expression level detected by qRT-PCR using random primers is the sum of the *Hist2h2aa2 *and *ASH2a *levels (Fig. 1). Because ASH2a has a unique sequence, the expression level of this antisense transcript can be detected separately. Observation using qRT-PCR indicated that the sum of the *Hist2h2aa2 *and *ASH2a *expression levels was always much higher than the level of *ASH2a *(Fig. 3). The difference between both C_T _values is more than 4. Thus, we estimated the expression of *Hist2h2aa2 *and *ASH2a *as that of *Hist2h2aa2 *in the following study. Along with cell cycle progression from S-phase, the expression of *Hist2h2aa2 *increased and peaked at 2 to 4 h, and then decreased (Fig. 4). After that, it increased again, but the expression level was lower than the S-phase peak. Thus, the expression is the highest in the middle of S-phase. On the other hand, that of *ASH2a *peaked at 1 h, and fell to basal level thereafter (Fig. 4). The rate of the increase of *ASH2a *RNA was lower than that of *Hist2h2aa2 *RNA.

## Discussion

An antisense transcript of histone H2a (ASH2a) was clearly expressed in three mouse cell lines, Hepa 1–6, 3T3, and LLC. This result strongly suggests that *ASH2a *is not regulated with tissue specificity. We checked the upstream regions of *ASH2a *and the promoter regions of mouse histone H2a genes. All promoters of replication-dependent histone H2a genes include CCAAT and TATA boxes [13]. On the other hand, the upstream region of *ASH2a *lacks such a structure (Fig. 1). This region has a G+C-rich sequence but lacks both CCAAT and TATA boxes (Fig. 1). Therefore, the regulation of *ASH2a *is strongly suggested to be different from those of replication-dependent histone H2a genes.

To check the synchronization of the cells, we compared the expression patterns of the replication-dependent histone gene *Hist2h2aa2 *and the replication-independent histone gene *H2afz *[16]. Hepa 1–6 cells used in the present study were well synchronized. In fact, the expression pattern of *ASH2a *was different from that of *Hist2h2aa2*. The amount of sense RNA was always much higher than that of *ASH2a *RNA at each time point. Three general functions of antisense transcripts have been identified: transcriptional interference, RNA masking, and dsRNA-dependent mechanisms, including RNA interference [17]. These functions are related to inhibition and/or degradation of sense RNAs. If ASH2a is related to the degradation of the sense RNA through dsRNA formation, *ASH2a *would be expressed when the sense RNA decreases. However, *ASH2a *is expressed during the early increase of the sense RNA.

On the other hand, ASH2a could hybridize not only to the *Hist2h2aa2 *transcript, but also to the transcripts of the other histone H2a-coding genes, because of the high similarity of protein coding sequences. A recent article showed that a small modulatory dsRNA can function as an activator of related genes [18], and the mechanism of action appears to be mediated through a dsRNA/protein interaction, rather than through siRNA or miRNA. Interestingly, ASH2a includes not only a sequence complementary to that of the *Hist2h2aa2 *transcript, but also a sequence complementary to the promoter region of *Hist2h2aa2*. Additional work is needed to elucidate the function of the ASH2a-related dsRNA.

Experiments using the high-density oligonucleotide arrays show that a large population of noncoding RNAs are expressed and regulated by similar molecular mechanisms to those involved in the control of protein-coding RNAs [19] and that many transcripts appear to be at very low abundance [20]. It is so important for genome research to elucidate the functions and regulation of noncoding RNAs and antisense RNAs at very low abundance such as ASH2a.

## Materials and Methods

### Cell lines

Murine hepatoma cell line Hepa 1–6, fibroblast cell line Flp-In-3T3 (Invitrogen), and lung carcinoma cell line LLC were cultured in DMEM supplemented with 10% fetal calf serum.

### Cell cycle synchronization

Hepa 1–6 cells were synchronized at the end of G1 phase by the addition of thymidine-hydroxyurea. The cell cycle arrest was released by washing out the thymidine-hydroxyurea, then the cells were harvested at intervals of 1 h from 0 h to 12 h.

### RT-PCR

Total RNA fractions extracted from mouse cells were pre-treated with DNase I and used for RT-PCR. Reaction mixture containing the RNA (approximately 0.5 μg) and the strand-specific primer (3.3 pmol) or random hexamer primers was denatured at 70°C, and then reverse-transcription reaction was done with Superscript III (Invitrogen) according to the manual. Then the cDNA was amplified by PCR under the condition of 35 or 40 cycles of 30 s at 95°C, 30 s at 54°C, and 1 or 2 min at 72°C. The sequences of primers are shown in Fig. 1. For quantitative RT-PCR (qRT-PCR), approximately 12.5 ng of total RNA was used for reverse transcription followed by PCR amplification with primers qF1 and R1, or qF2 and R2 (Fig. 1) in reaction mixture containing SYBR premix Ex *Taq *(Takara) in an ABI PRISM 7700 sequence detection system (Applied Biosystems). The PCR conditions were an initial step of 30 s at 95°C, followed by 40 cycles of 5 s at 95°C and 30 s at 60°C. Expression was assessed by evaluating threshold cycle (C_T_) values. The relative amount of expressed RNA was calculated using Livak and Schmittgen's method [21]. Quantification of GAPDH (glyceraldehyde-3-phosphate dehydrogenase) mRNA (primers 5'-TGTGTCCGTCGTGGATCTGA-3' and 5'-CCTGCTTCACCACCTTCTTGA-3'; product size 76 bp) was used as a control for data normalization. The qRT-PCR analyses were performed four times.

## Authors' contributions

HN designed this study, carried out the molecular biological studies. YT carried out synchronization of cells and quantitative PCR. YO and YH participated in the design of this study.
